# An Integrated Platform for the Comprehensive Analysis of Host‐Fungal Interactions in the Murine Tongue

**DOI:** 10.1002/cpz1.70473

**Published:** 2026-09-25

**Authors:** Julia Lagler, Mathieu Courivaud, Johnny Bonnardel, Salomé LeibundGut‐Landmann

**Affiliations:** ^1^ Section of Immunology, Vetsuisse Faculty University of Zürich Zürich Switzerland; ^2^ Institute of Experimental Immunology University of Zürich Zürich Switzerland; ^3^ Centre d'Immunologie Marseille‐Luminy, Aix‐Marseille University, INSERM, CNRS Marseille France; ^4^ Medical Research Council Centre for Medical Mycology at the University of Exeter Department of Biosciences, Faculty of Health and Life Sciences Exeter UK; ^5^ These authors contributed equally

**Keywords:** Candida albicans, oral fungal colonization, homeostatic immunity

## Abstract

This protocol describes a mouse model of *Candida albicans* oral colonization that establishes stable, long‐term fungal persistence in the oral cavity, thereby recapitulating key features of human commensal colonization, including the induction of sustained adaptive antifungal immunity. The model enables the analysis of host‐fungal interactions across multiple experimental readouts. Procedures are provided for isolation of single‐cell suspensions from the whole tongue, the tongue epithelium, and the draining lymph nodes for flow cytometric analysis, supporting both enumeration and extended functional characterization of immune cell populations. Antigen‐specific T cell responses can be assessed by re‐stimulation of isolated T cells with *C. albicans*‐pulsed dendritic cells prior to staining and analysis. Additional methods include quantification of fungal burden in tongue tissue, histological assessment of fungal localization and immune cell infiltration, and isolation of high‐quality RNA using an optimized extraction workflow compatible with RT‐qPCR and RNA sequencing. Collectively, these protocols provide a flexible and integrated platform for studying immune responses and host‐fungal interactions during persistent oral colonization. © 2026 The Author(s). *Current Protocols* published by Wiley Periodicals LLC.

**Basic Protocol 1**: Oral colonization of mice with *Candida albicans*

**Basic Protocol 2**: Preparation of single‐cell suspensions from the murine whole tongue, tongue epithelium, and draining lymph nodes

**Basic Protocol 3**: Re‐stimulation of cLNs and tongue T cells for qualitative and quantitative assessment of T cell effector functions

**Basic Protocol 4**: Flow‐cytometry‐based analysis of tongue immune cell populations

**Support Protocol 1**: Evaluation of fungal burden in the tongue of *C. albicans*‐colonized mice

**Support Protocol 2**: Visualization of tissue integrity, tissue inflammation, and fungal localization by histology

**Support Protocol 3**: Extraction of total RNA from the tongue of *C. albicans*‐colonized mice for RNA sequencing and RT‐qPCR

## INTRODUCTION

*Candida albicans* is a common member of the human microbiota modulating diverse physiological host processes including barrier integrity and host defenses (Shao et al., 2022). In susceptible hosts, however, *C. albicans* exerts pathological effects and causes infections, including common oral and vaginal candidiasis (Denning et al., 2018; Vila et al., 2020) as well as more severe systemic infections associated with a high mortality rate (Denning, 2024). *C. albicans* also contributes to noninfectious diseases by exacerbating inflammatory conditions such as Crohn's disease and liver pathologies (Harberts & Schnabl, 2026; Li et al., 2019). In healthy individuals, the oral cavity represents a prominent niche for *C. albicans*, which is readily detected in the oral microbiota (Ghannoum et al., 2010). The stratified epithelium of the oral mucosa, with its terminally differentiated outermost layer of anucleated cells, provides a sheltered space in which the fungus can from physical, chemical, and immune stresses that would otherwise compromise oral colonization (Frois‐Martins et al., 2025).

Most of what we currently know about *C. albicans*‐host interactions in the oral cavity was acquired from *in vitro* fungus‐host interaction assays and from murine models of oropharyngeal candidiasis. During experimental oropharyngeal candidiasis, in which highly virulent strains of *C. albicans* (e.g., SC5314) are administered sublingually to wild‐type C57BL/6 mice (Solis & Filler, 2012), the fungus induces a strong inflammatory response in the oral mucosa and is rapidly cleared (Conti et al., 2009; Gladiator et al., 2013). If mice are immunosuppressed (e.g., with corticosteroids) before infection, sublingual *C. albicans* infection results in excessive fungal growth, tissue invasion, and dissemination accompanied by massive weight loss (Solis & Filler, 2012). Although these models shed some light on the molecular and cellular events occurring during acute *C. albicans* oral infection, they do not reflect the most common situation of *C. albicans*‐host interaction, i.e., harmless colonization of the healthy host—a condition under which *C. albicans* exists in a commensal relationship with the host, contributing to tissue homeostasis.

Several approaches have been pursued to establish long‐lasting *C. albicans* colonization in mice, allowing the fungus‐host interplay and the antifungal host response to be studied over time. The most commonly used *C. albicans* colonization model consists of administering *C. albicans* by gavage to mice previously treated with antibiotics to limit colonization resistance in the gastrointestinal tract (Fan et al., 2015; Ost et al., 2021; Tso et al., 2018; Witchley et al., 2019). Under these conditions, *C. albicans* strains defective in filamentation and hyphae‐associated gene expression were found to be the most efficient colonizers, which in turn led to the notion that hyphae and other virulence factors prevent colonization (Bohm et al., 2017; Witchley et al., 2019). This paradigm has recently been challenged by gastrointestinal colonization of eubiotic mice (i.e., mice with a normal SPF microbiota that have not been antibiotically treated; Liang et al., 2024). Vaginal colonization with *C. albicans* likewise depends on preconditioning mice with estrogen to simulate a pseudoestrus state receptive to the fungus (Cassone & Sobel, 2016). This model mimics vulvovaginal candidiasis in humans, which is characterized by a strong neutrophil response (MacAlpine & Lionakis, 2024). Together, these models have important limitations as antibiotic or hormonal treatments dysregulate the microbial and host status and thus do not faithfully mimic the homeostatic state of fungal colonization in naturally colonized humans.

We have established a mouse model of *C. albicans* colonization that is independent of any preconditioning of the colonized animals (Schonherr et al., 2017). The model relies on the sublingual administration of a low‐virulence isolate of *C. albicans* to wild‐type C57BL/6 mice, which establishes stable colonization of the oral mucosa, including the tongue, for several months and in some cases even over a year, without causing noticeable tissue damage or inflammation. The model makes it possible to study the crosstalk of *C. albicans* with the host during steady state, including the fungal determinants of oral colonization and the antifungal immune response that keeps the fungus in check over time (Frois‐Martins et al., 2025; Kirchner & LeibundGut‐Landmann, 2021; Kirchner et al., 2019; Lemberg et al., 2022; Schonherr et al., 2017). The immune response against *C. albicans* colonization is characterized by a noninflammatory type 17 response with IL‐17‐producing CD4^+^ T cells that reside within the tissue for prolonged periods of time (Kirchner & LeibundGut‐Landmann, 2021), reminiscent of what has been described in naturally colonized humans (Park et al., 2018), while other immune cell populations also contribute to mounting a protective response. Although the model does not fully recapitulate the complexity of the factors modulating *C. albicans*‐host interactions in the human oral mucosa, such as diet, age, or microbiota composition, it is highly valuable for dissecting mechanisms of fungal colonization. The model also makes it possible to probe how the equilibrium between the fungus and host is disrupted upon interference with antifungal immunity (Frois‐Martins et al., 2026), as may occur during the course of life of a naturally colonized individual, for instance as a consequence of induced immunosuppression or immunotherapy (Lionakis et al., 2023).

This article provides protocols for the oral colonization of mice with *C. albicans* (Basic Protocol 1), the preparation of tongue and draining lymph node single‐cell suspensions (Basic Protocol 2), and the subsequent flow cytometric analysis of tongue immune cell populations (Basic Protocols 3 and 4). It also includes support protocols for evaluating the fungal load in the colonized tongue (Support Protocol 1), for histological analysis of the fungus and the antifungal response in the colonized tongue (Support Protocol 2), and for reverse transcription quantitative PCR (RT‐qPCR)‐based analysis of host and fungal transcripts in the colonized tongue (Support Protocol 3).

*NOTE*: Animal experimentation is regulated by national law. Ensure compliance with all national and local guidelines and regulations. All protocols involving animals must be reviewed and approved by the appropriate Animal Care and Use Committee and must follow regulations for the care and use of laboratory animals. Appropriate informed consent is necessary for obtaining and use of human study material.

*CAUTION: Candida albicans* is classified as a Biosafety Level 2 (BSL‐2) pathogen. Follow all appropriate guidelines and regulations for the use and handling of pathogenic microorganisms.

## Basic Protocol 1 ORAL COLONIZATION OF MICE WITH *CANDIDA ALBICANS*

This protocol describes the preparation of a viable inoculum of *C. albicans* for establishing oral fungal colonization in mice. The procedure ensures consistent, reproducible association of mice with *C. albicans* under noninflammatory conditions and induction of homeostatic antifungal immunity.

### Materials

*Candida albicans* strain 101 or alternative low‐damage‐inducing strain (Frois‐Martins et al., 2026; Schonherr et al., 2017), frozen stock
YPD agar plates (see recipe)
YPD liquid medium (see recipe)
Phosphate‐buffered saline (PBS), sterile (BioConcept, cat. no. 3‐05F39‐I, or equivalent)
C57BL/6JRj mice (Janvier)
Ketamine/xylazine mix (see recipe)
Eye drops (Viscotears, 2 mg/g Carbomer 980; Bausch + Lomb, cat. no. 1551535, or equivalent)
Lactated Ringer's solution, sterile (saline for intravenous injection; Fresenius, cat. no. 3671261, or equivalent)

Laminar flow hood (Faster or equivalent)
Microbiological incubator, set at 30°C (Heraeus or equivalent)
Shaking incubator, set at 30°C, 180 rpm (Infors or equivalent)
Inoculation loops, sterile (Sarstedt, cat. no. 86.1562.050, or equivalent)
12‐ml round‐bottom polypropylene tubes with caps, sterile (Falcon, cat. no. 352057, or equivalent)
Pipet controller (Integra Pipetboy 2 or equivalent)
Cuvettes (Greiner, cat. no. 7001356)
Spectrophotometer (Jenway 6300 or equivalent)
100‐ml Erlenmeyer flask with stopper, sterile (Huberlab, cat. no. 9.0460.24, or equivalent)
1.5‐ and 2‐ml Eppendorf Safe‐Lock microcentrifuge tubes, sterile (Eppendorf, cat. nos. 7001817 and 0030 123.344, or equivalent)
Microcentrifuge (Eppendorf model 5415D or equivalent)
Vortex‐Genie 2 (Scientific Industries or equivalent)
2.5‐mg cotton balls, sterile (Omnident, cat. no. 6049376, or equivalent)
0.5‐ml, 30‐G × 12‐mm syringes, sterile (Omnicam 50, B. Braun, cat. no. 9151125, or equivalent)
Heating pad (Eickemeyer, cat. no. 648048, or equivalent)
Two pairs of curved dressing forceps (B. Braun Medical AG, cat. no. BD312R, or equivalent)

#### Preparation of a *C. albicans* inoculum for oral colonization of mice

1 Streak a loopful of *C. albicans* from a glycerol stock (stored at −80°C) onto YPD agar plates and incubate for 24 hr at 30°C.
  C. albicans growing on YPD agar can be stored at 4°C for up to 14 days before a new agar plate should be prepared from frozen stocks.
2 Set up a fungal pre‐culture by inoculating 5 ml YPD medium in a 12‐ml polypropylene tube with a loopful of *C. albicans* from YPD agar. Vortex and incubate for 6‐8 hr at 30°C in a shaking incubator (180 rpm).
3 Determine the optical density of this pre‐culture (OD_600_) using a spectrophotometer.
4 Inoculate 10 ml fresh YPD medium in a 100‐ml Erlenmeyer flask with an aliquot of the pre‐culture (step 2) to achieve OD_600_ = 0.1. Incubate the culture for 12‐18 hr at 30°C in a shaking incubator (180 rpm).
5 Quickly swirl the Erlenmeyer flask, and then remove 500 µl from the culture and transfer to a 2‐ml Safe‐Lock Eppendorf tube.
6 Centrifuge for 1 min at 10,000 × *g*, room temperature, and discard the supernatant.
7 Perform three washes, in each case resuspending the fungal pellet in 1 ml PBS, vortexing briefly to fully suspend the fungal cells, centrifuging as in step 6, and discarding the supernatant.
8 Resuspend the fungal pellet in 1 ml PBS and determine the cell concentration by measuring OD_600_ using a spectrophotometer. 1 OD_600_ = 10^7^ yeast cells/ml.
9 Adjust the concentration of the fungal suspension to 5 × 10^7^ yeast cells/ml with PBS.
10 Aliquot 100 µl fungal suspension into 1.5‐ml Safe‐Lock Eppendorf tubes each containing a 2.5‐mg sterile cotton ball. Prepare one tube per mouse to be colonized.
  The cotton balls will absorb ∼50 µl of the fungal suspension, which corresponds to ∼2.5 × 10^6^ yeast cells; thus, the infection dose is ∼2.5 × 10^6^ yeast cells per mouse. The concentration of the inoculum can be adjusted if a higher or lower inoculation dose is desired.

#### Oral association of mice with *C. albicans*

11 Anesthetize mice by intraperitoneal injection of 14 µl per gram body weight (µl/g) of ketamine/xylazine mix diluted in sterile PBS.
  Injection of 14 µl ketamine/xylazine mix per gram of body weight corresponds to 91 mg/kg ketamine and 18.2 mg/kg xylazine. Prepare the mix freshly on the day of use.
  With some experience, up to 20‐25 mice can be treated in parallel.
  We typically use C57BL/6JRj mice from Janvier at 6‐8 weeks of age (females or males).
12 Check reflexes to ensure deep anesthesia.
13 Place the sedated mice on a heating pad at 35‐37°C.
14 Apply vitamin A ointment to the eyes of the mice to avoid desiccation.
15 Position the mice on their backs, with their heads facing towards the front side of the pad.
16 Using a pair of forceps, carefully open the mouth of the first mouse and lift the tongue towards the palate. Using a second pair of forceps, carefully place a saturated cotton ball sublingually (between the tongue and mandible).
  To ensure a homogeneous inoculum, gently remix the fungal suspension with the cotton ball immediately before removing the cotton ball from the Eppendorf tube.
17 Close the mouth of the mouse and turn it on its abdomen.
18 Perform steps 15‐17 for the remaining mice, minimizing the time interval between animals.
19 At 30‐40 min after the initial injection of ketamine/xylazine, inject another 2.5 µl/g of the ketamine/xylazine mix—corresponding to 16.25 mg/kg ketamine and 3.25 mg/kg xylazine—to keep the mice sedated for up to 90 min. Closely monitor the mice and reapply vitamin A ointment if their eyes start looking dry.
20 At least 75 min after placing the cotton ball, remove the cotton ball and dispose of it in a biosafety‐compliant manner.
21 Inject 200 µl sterile Ringer's lactate subcutaneously into the loose skin over the neck.
22 Monitor the mice until they recover from anesthesia and place them back in their cage.
  After oral colonization with a low‐damage‐inducing strain of C. albicans such as 101, mice typically show a mild and transient body weight loss of <5% during the first 2‐3 days and rapidly regain weight thereafter. There are usually no other adverse effects such as reduced grooming, mobility, food intake, and/or postural changes. No lesions are macroscopically visible on the tongue surface after colonization.

## Basic Protocol 2 PREPARATION OF SINGLE‐CELL SUSPENSIONS FROM WHOLE TONGUE, TONGUE EPITHELIUM, AND DRAINING LYMPH NODES

This protocol describes the preparation of single‐cell suspensions from whole tongue, tongue epithelium, and draining lymph nodes (cervical lymph nodes) of *Candida albicans*‐colonized mice for flow cytometry analysis. The single‐cell suspension from the tongue epithelium consists primarily of epithelial cells and immune cells involved in the antifungal response in the epithelium, whereas the suspension prepared from the whole tongue contains a substantial fraction of non‐immune cells, including fibroblasts and muscle cells.

### Materials

Mice orally colonized by *C. albicans* (Basic Protocol 1)
MACS buffer, sterile (see recipe), 4°C
Phosphate‐buffered saline (PBS), sterile (BioConcept, cat. no. 3‐05F39‐I, or equivalent), 4°C
Collagenase I/DNase I digestion mix for cLNs (see recipe), 20 µl per sample
Collagenase IV/DNase I digestion mix for whole tongue (see recipe), 20 µl per sample
Dispase II digestion mix for tongue epithelium (see recipe), 500 µl per sample
Collagenase II/DNase I digestion mix for tongue epithelium (see recipe), 1 ml per sample; see recipe
RPMI 1640 without l‐glutamine (Life Technologies, cat. no. 21875034, or equivalent)
Complete R10 medium (see recipe)

Laminar flow hood (Faster or equivalent)
2‐ml Eppendorf Safe‐Lock microcentrifuge tubes (Eppendorf, cat. no. 0030 123.344, or equivalent)
0.5 × 16‐mm, 25‐G needles, sterile (BD Microlance 3, cat. no. 300600, or equivalent)
20‐ml syringes (B. Braun Medical AG, cat. no. 7001173, or equivalent)
Two pairs of curved dressing forceps (B. Braun Medical AG, cat. no. BD312R, or equivalent)
Dissecting scissors (Karl Hammacher GmbH, cat. no. 6075888, or equivalent)
Type 20 surgical blades, sterile (Swann Morton, cat. no. 859221, or equivalent)
Vortex‐Genie 2 (Scientific Industries or equivalent)
Tabletop centrifuge with swing‐out rotor (Heraeus Megafuge 16R or equivalent)
Heating block (Eppendorf Thermomixer R or equivalent) or water bath, set to 37°C
Pipet controller (Integra Pipetboy 2 or equivalent)
70‐ and 100‐µm‐pore‐size cell strainers, sterile (BD/Falcon, cats. no. 352350 and 734‐0004, or equivalent)
50‐ml Falcon tube, sterile (Sarstedt, cat. no. 7004175, or equivalent)
92 × 16‐mm petri dishes, sterile (Sarstedt, cat. no. 82.1473, or equivalent)
Vacuum pump (Vacuubrand, BVC Professional, or equivalent)
1‐ml syringes (Injekt‐F 1 ml, B. Braun AG, cat. no. 7000323, or equivalent)
30‐G × 12‐mm, 0.5‐ml syringes, sterile (B. Braun Omnicam 50, cat. no. 9151125, or equivalent)
Precision balance reading to four decimal places (0.0001 g)

#### Collection of the cervical lymph nodes (cLN) and preparation of a single‐cell suspension

1 Prepare one 2‐ml Safe‐Lock Eppendorf tube per mouse containing 1 ml MACS buffer. Keep on ice.
2 Euthanize mice sequentially by CO_2_ inhalation. Perform transcardiac perfusion with 15‐20 ml cold PBS to flush systemic blood and remove erythrocytes and circulating immune cells from the tissue.
  If tissue is collected for fungal load enumeration (Support Protocol 1), histology (Support Protocol 2), or RNA isolation (Support Protocol 3), no transcardiac perfusion is required, and another method may be used to ascertain the death of the animal.
  Transcardiac perfusion must be performed shortly after euthanasia.
3 Place the mouse in dorsal recumbency. Using scissors, make a shallow midline incision from the thorax toward the mandible, with lateral extensions toward both forelimbs. Separate the skin from the underlying tissue layer and pin it laterally to expose the cLNs adjacent to the salivary glands. Typically, two to four nodes (submandibular and superficial parotid lymph nodes) are found per side.
4 Remove the cLNs and place them in the tube with MACS buffer. Keep on ice.
  Steps 5‐14, 20‐34, and 36‐49 should be performed aseptically if the cells will be used for in vitro re‐stimulation assays.
5 After collecting cLNs from all animals, add 20 µl collagenase I/DNase I digestion mix for cLNs to the tubes containing the cLNs in MACS buffer.
6 Vortex briefly to mix and centrifuge quickly.
7 Incubate in a heating block at 37°C (no shaking) for 20 min.
8 Place tubes on ice.
9 Filter the digested cLNs through a 70‐µm strainer into a 50‐ml Falcon tube.
10 Mechanically dissociate the tissue by pressing it through the strainer with the plunger of a 1‐ml syringe.
11 Rinse the strainer with 10 ml cold MACS buffer and continue dissociating any remaining tissue.
12 Rinse the strainer again with an additional 10 ml cold MACS buffer.
13 Centrifuge for 5 min at 560 × *g*, 4°C, and discard the supernatant.
14 Resuspend the pellet in:
  - 1 ml complete R10 medium in preparation for T cell re‐stimulation and intracellular staining (see Basic Protocol 3); or
  - 1 ml MACS buffer in preparation for cell surface staining (see Basic Protocol 4).

#### Tongue collection and preparation of a single‐cell suspension from the whole tongue

15 Prepare one 2‐ml Safe‐Lock Eppendorf tube per mouse containing 1 ml PBS. Keep on ice.
16 Euthanize, perfuse, and dissect the mice as described for cLN harvesting (steps 2 and 3).
  If both tissues are required, it is recommended that the cLNs be collected before the tongue is removed.
17 Cut along the labial commissure of the mouth and through the mandibular joint to expose the tongue. Remove the apical portion of the mandible, including the lower incisors, with a horizontal cut.
18 Secure the tongue gently with forceps. Using scissors, cut along the ventral side until you reach the base of the tongue.
  Handle the tongue with care, to preserve tissue integrity, if it is to be used for histology (Support Protocol 2).
19 Detach the tongue with one horizontal cut at the base, transfer immediately to the prepared PBS tube, and place tube on ice.
20 Place the tongue in a sterile petri dish and cut it longitudinally into two halves.
21 Position the cut side up and scrape off the muscle layer four or five times using a scalpel blade.
  Make a small longitudinal incision at the apex of the tongue to improve stability during scraping. Avoid excessive scraping to prevent disruption of the epithelial and subepithelial tissue.
  Optionally: Determine the weight of the tongue by taking the weight of the tubes before and after adding the tongue. Knowing the weight of the tongue will make it possible to normalize cell counts acquired by flow cytometry to the weight.
22 Cut the scraped half of the tongue longitudinally into two equal pieces and transfer each piece into separate 2‐ml Safe‐Lock Eppendorf tubes containing 1 ml PBS.
  The other half tongue may be processed the same or used for fungal load enumeration (Support Protocol 1), histology (Support Protocol 2), or RNA isolation (Support Protocol 3).
  For T cell re‐stimulation followed by intracellular staining (Basic Protocol 3), it is recommended to use >½ tongue.
  One‐third to one‐half of a tongue is enough for cell surface staining (Basic Protocol 4).
23 Using scissors, finely mince the tissue inside the tube.
24 Add 20 µl collagenase IV/DNase I digestion mix for whole tongue to the minced tissue.
25 Vortex briefly to mix and centrifuge quickly.
26 Incubate in a heating block at 37°C for 45 min.
27 Place tubes on ice.
28 Transfer the digested tongue suspension into a 70‐µm cell strainer (placed on a 50‐ml Falcon tube) using a 1‐ml pipet with a cut tip.
29 Mechanically dissociate the tissue by pressing it through the strainer with the plunger of a 1‐ml syringe.
30 Rinse the strainer with 10 ml cold MACS buffer, and continue dissociating any remaining tissue until fully dissociated.
31 Rinse the strainer again with additional 10 ml cold MACS buffer.
32 Centrifuge for 15 min at 560 × *g*, 4°C.
33 Carefully discard the supernatant without disturbing the pellet.
  This is best done by gently tilting the tube at a 45° angle and aspirating with a vacuum pump, rather than by decanting.
34 Resuspend the pellet in:
  - 350 µl complete R10 medium for T cell re‐stimulation in preparation for intracellular staining (see Basic Protocol 3); or
  - 1 ml MACS buffer in preparation for cell surface staining (see Basic Protocol 4).
  - *The tongue pellet has a sticky consistency and needs to be resuspended well by pipetting up‐and‐down multiple times*.

#### Preparation of a single‐cell suspension from the tongue epithelium

**Figure 1:**
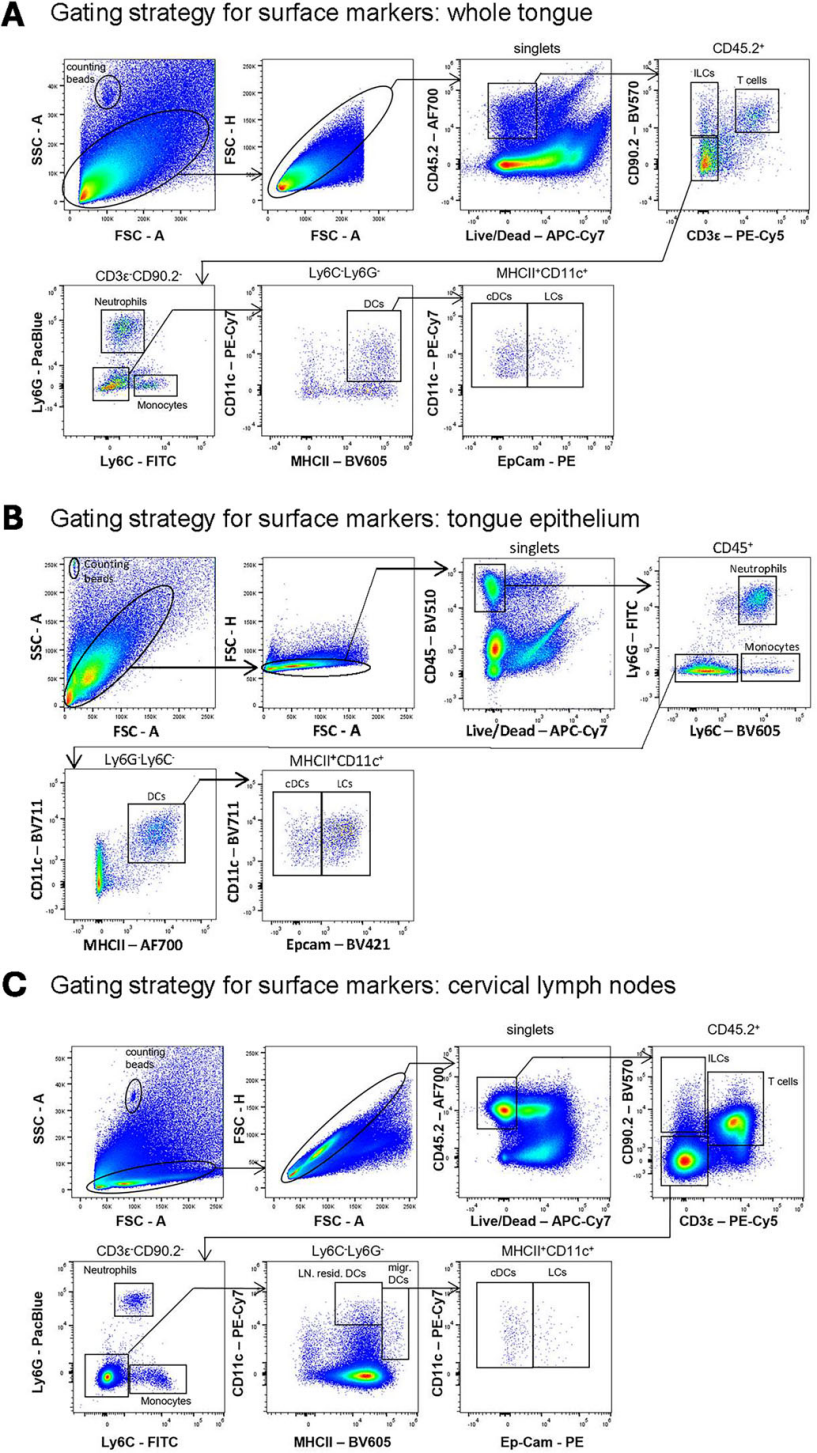
Gating strategy for myeloid cells in whole tongue, tongue epithelium, and cervical lymph nodes of *C. albicans*‐colonized mice. Representative flow plots showing myeloid cell types including monocytes (Ly6C⁺Ly6G^−^), neutrophils (Ly6G⁺Ly6C^−^), DCs (CD11c⁺MHCII⁺EpCAM^−^), and Langerhans cells (LCs; CD11c⁺MHCII⁺EpCAM⁺) in the whole tongue (**A**), tongue epithelium (**B**), and cervical lymph nodes (**C**).

35 Collect the tongue as described in steps 16‐19.
36 Transfer the whole tongue into a 2‐ml Safe‐Lock Eppendorf tube containing 1 ml RPMI 1640 without l‐glutamine.
  For preparing a single‐cell suspension of the tongue epithelium, the whole tongue is used.
37 Inject ∼500 µl dispase II digestion mix into the tongue using a sterile 0.5‐ml syringe.
  First inject at the tip of the tongue, and then insert the needle at the dorsal root to fill the remaining tissue.
  Adequate delivery is indicated by swelling of the tongue to approximately twice its original volume.
38 Transfer the tongue to a 2‐ml Safe‐Lock Eppendorf tube containing 1 ml prewarmed RPMI medium (without additives).
39 Incubate in a heating block at 37°C for 40 min (no shaking).
40 After digestion, transfer the tongue onto a sterile petri dish. Using forceps, grasp the epithelium at the posterior dorsal surface and peel it away from the underlying tissue.
  If the digestion worked well, the epithelium should come off in one piece.
41 Transfer epithelial tissue to a 1.5‐ml Safe‐Lock Eppendorf tube containing 500 µl collagenase II/DNase I digestion mix for whole tongue.
  Optionally: Determine the weight of the epithelium by taking the weight of the tubes before and after adding the epithelium. Knowing the weight of the epithelium will allow normalizing cell counts acquired by flow cytometry to the weight.
42 Using scissors, finely mince the tissue inside the tube and then add an additional 500 µl collagenase II/DNase I digestion mix.
43 Incubate in a heating block at 37°C, 1100 rpm, for 25 min.
  Mix the epithelial fragments in the digestion mix by up‐and‐down pipetting with a 1‐ml pipet every 5 min. To minimize loss of epithelial tissue on the wall of the pipet tip, it is advisable to prewet the pipet tip with the digestion mix.
44 Transfer the digested tongue epithelium onto a 70‐µm strainer placed on a 50‐ml tube and prewetted with 3 ml cold MACS buffer.
45 Mechanically dissociate the tissue by pressing it through the strainer with the plunger of a 1‐ml syringe.
46 Rinse the strainer with 10 ml cold MACS buffer.
47 Centrifuge for 5 min at 560 × *g*, 4°C.
48 Carefully discard the supernatant without disturbing the pellet.
  This is best done by gently tilting the tube at a 45° angle and aspirating with a vacuum pump, rather than by decanting.
49 Resuspend the cell pellet in:
  - 300 µl complete R10 medium in preparation for T cell re‐stimulation followed by intracellular staining (see Basic Protocol 3); or
  - 1 ml MACS buffer in preparation for cell surface staining (see Basic Protocol 4).
    Typically, single‐cell suspensions prepared from whole tongue are very heterogeneous, with large numbers of epithelial and stromal cells in addition to immune cells. CD45^+^ leukocytes, including myeloid and lymphoid cell populations, can reliably be detected, although their frequencies within the total number of acquired events are relatively low (Fig. 1A). The presence of substantial amounts of intrinsically autofluorescent dead cells and debris in the single‐cell suspension requires extra care when setting the parameters for flow cytometric data acquisition and analysis.
    Single‐cell suspensions of tongue epithelium are enriched for epithelial cells and immune cells associated with the epithelium, including those involved in the antifungal response to C. albicans colonization. The epithelial cell preparations are typically cleaner than single‐cell suspensions prepared from the whole tongue, with lower autofluorescence and thus less background signal in flow cytometry (Fig. 1B).
    cLN processing typically yields a high‐quality single‐cell suspension characterized by high cell viability and low amounts of autofluorescent cells and debris (Fig. 1C). Compared to tongue‐derived samples, cLN suspensions are generally less challenging to analyze by flow cytometry.

## Basic Protocol 3 RE‐STIMULATION OF cLNs AND TONGUE T CELLS FOR QUALITATIVE AND QUANTITATIVE ASSESSMENT OF T CELL EFFECTOR FUNCTIONS

This protocol describes how cLNs and tongue T cells are stimulated *ex vivo* to elicit antigen‐specific cytokine production by effector and memory T cells to be assessed by flow cytometry. Alternatively, polyclonal stimulation using PMA/ionomycin may be used; in that case, begin the procedure at step 11b.

### Materials

DC1940 cells (immortalized mouse dendritic cell line; Fuertes Marraco et al., 2012)
Complete IMDM medium (see recipe), prewarmed to 37°C
5 mM EDTA (see recipe)
Complete R10 medium (see recipe)
Trypan blue solution (see recipe)
cLN cells, whole tongue, or tongue epithelial cells (Basic Protocol 2)
Heat‐killed *C. albicans*, 5 × 10^7^ yeast cells/ml (same strain as used for colonization of mice; see recipe) or PMA/ionomycin (see recipe)
Brefeldin A solution (see recipe)

Laminar flow hood (Faster or equivalent)
Pipet controller (Integra Pipetboy 2 or equivalent)
75‐cm^2^ cell culture flask, sterile (TPP, cat. no. 7000199, or equivalent)
Cell culture incubator (HERAcell 150i or equivalent) set to 37°C and 5% CO_2_
50‐ml Falcon tubes, sterile (Sarstedt, cat. no. 7004175, or equivalent)
Tabletop centrifuge with swing‐out rotor (Heraeus Megafuge 16R or equivalent)
Neubauer counting chamber
Water bath, 37°C
96‐well flat‐bottom cell culture plate (TPP, cat. no. 7000209 or equivalent)

#### Seeding dendritic cells (DCs) for antigen‐specific T cell re‐stimulation

1 Expand DC1940 cells in 75‐cm^2^ flasks with 12 ml complete IMDM medium and grow for at least 24‐48 hr at 37°C, 5% CO_2_.
  When initiating the culture from a cryopreserved vial, thaw and seed 4 × 10^6^ cells into a 25‐cm^2^ flask and then transfer into a 75‐cm^2^ flask. Passage cells at least once after thawing before use. For routine maintenance, dilute cells 1:10 from an almost confluent flask; under these conditions, cells are typically ready for use after 2‐3 days. Healthy DC1940 cells are round, adhere to the flask surface, and display several dendrite‐like protrusions.
2 Aspirate medium and add 5 ml ice‐cold 5 mM EDTA to harvest cells.
3 Incubate 5 min at room temperature or until cells detach.
  Trypsinization is unnecessary as DC1940 cells are loosely adherent.
4 Add 10 ml prewarmed complete IMDM medium and transfer the cell suspension to a 50‐ml Falcon tube.
5 Centrifuge 5 min at 560 × *g*, room temperature.
6 Discard the supernatant and resuspend the cell pellet in 10 ml prewarmed complete R10 medium.
7 Determine the cell concentration using a cell counting chamber and trypan blue to discriminate nonviable cells.
8 Adjust the cell density to 1 × 10^6^/ml in prewarmed complete R10 medium.
9 Seed 100 µl = 10^5^ DC1940 cells per well in flat‐bottom 96‐well cell culture plates.
  - cLNs: prepare 3 wells per sample;
  - Whole tongue: prepare 4 wells per sample;
  - Tongue epithelium: prepare 3 wells per sample.
10 Incubate at 37°C for at least 1 hr, to give the cells time to adhere to the bottom of the plate, before proceeding to the next step.
  If DC1940 cells are seeded on the day before T cell re‐stimulation, seed only 0.8 × 10^5^ in 100 µl complete R10 medium per well and incubate at 37°C, 5% CO_2_ overnight.
11a *For antigen‐specific re‐stimulation* *of cLN and tongue cells using DCS pulsed with heat‐killed C. albicans*: Add 5 µl of heat‐killed *C. albicans* (1 × 10^7^/ml, diluted from stock solution) per well (= 5 × 10^4^ yeast cells per well).
11b *For polyclonal re‐stimulation of cLN and tongue cells*: As an alternative to antigen‐specific restimulation, add 100 µL PMA/ionomycin solution per well to a round‐bottom 96‐well cell culture plate (final concentration: 50 ng/ml PMA; 500 ng/ml ionomycin).
12 Add cLN or tongue cells to the 96‐well plate containing either fungus‐pulsed DC1940 cells or PMA/ionomycin:
  - cLN cells from Basic Protocol 2, step 14: 100 µl cLN single‐cell suspension (3 wells/sample);
  - total tongue cells from Basic Protocol 2, step 34: 100 µl whole tongue single‐cell suspension (4 wells/sample); or
  - tongue epithelial cells from Basic Protocol 2, step 49: 100 µl epithelial single‐cell suspension (3 wells/sample).
    Tongue single‐cell suspensions are slightly sticky and should therefore be resuspended thoroughly by repeated up‐and‐down pipetting before being added to the DC1940 cells.
13 Incubate for 1 hr at 37°C, 5% CO_2_.
14 Add 20 µl brefeldin A solution per well (to 5 µg/ml final) to inhibit protein transport from the endoplasmic reticulum to the Golgi apparatus.
15 Incubate for 4 hr at 37°C, 5% CO_2_.
16 Proceed immediately to the staining of the cells for flow cytometry (Basic Protocol 4).

## Basic Protocol 4 FLOW CYTOMETRY‐BASED ANALYSIS OF TONGUE AND cLN IMMUNE CELL POPULATIONS

This protocol describes how immune cells isolated from cLNs, whole tongue, or tongue epithelium are stained for flow cytometric analysis with anti‐mouse monoclonal antibodies targeting cell surface and intracellular markers. The enclosed staining panels (Table 1 for T cells, Tables 2 and 3 for myeloid cells) were designed for data acquisition on a 10‐ to 11‐color CytoFLEX S flow cytometer to ensure broad accessibility, but these are provided only as examples and may readily be adapted (or expanded) according to the cell type(s) of interest to be analyzed and/or the flow cytometer available.

**Table 1:** Antibody Panel for Th17 Cells (Intracellular Cytokine Staining)^*a*^

**Antigen**	**Fluorochrome**	**Clone**	**Manufacturer**	**Cat. no**.	**Dilution**
IL‐22	PE	Poly5164	BioLegend	516404	1:100
CD103	PE‐Dazzle594	2E7	BioLegend	121429	1:200
CD3ε	PE‐Cy5	145‐2C11	BioLegend	100310	1:250
IL‐17A	PE‐Cy7	TC11‐18H10.1	BioLegend	506922	1:100
CD44	APC	IM7	BioLegend	103012	1:250
CD45.2	AF700	A20	BioLegend	110724	1:200
Viability dye, near‐IR	APC‐Cy7	–	eBioscience	65‐0865‐14	1:1000
CD90.2	BV570	30‐H12	BioLegend	105329	1:250
CD4	BV605	RM4‐4	BioLegend	105037	1:100

a IL‐22 and IL‐17A are intracellular antigens; the other antigens listed are surface antigens.

**Table 2:** Antibody Panels for Immune Subsets (Whole Tongue and cLNs)^*a*^

**Antigen**	**Fluorochrome**	**Clone**	**Manufacturer**	**Cat. no**.	**Dilution**
Ly6C	FITC	AL‐21	BD Biosciences	553104	1:200
Ep‐Cam	PE	G8.8	BioLegend	118206	1:250
CD3ε	PE‐Cy5	145‐2C11	BioLegend	100310	1:250
CD11c	PE‐Cy7	N418	BioLegend	117318	1:100
CD45.2	AF700	A20	BioLegend	110724	1:200
Viability dye, near‐IR	APC‐Cy7	–	eBioscience	65‐0865‐14	1:1000
Ly6G	PacBlue	1A8	BioLegend	127612	1:250
CD90.2	BV570	30‐H12	BioLegend	105329	1:250
MHCII	BV605	M5/114.15.2	BioLegend	107639	1:500

a All antigens listed are surface antigens.

**Table 3:** Antibody Panel for Myeloid Subsets (Tongue Epithelium)^*a*^

**Antigen**	**Fluorochrome**	**Clone**	**Manufacturer**	**Cat. no**.	**Dilution**
Ly6G	FITC	1A8	BioLegend	127625	1:300
Viability dye near‐IR	eF780	–	eBioscience	65‐0865‐14	1:4000
Ep‐cam	eF450	G8.8	eBioscience	48‐5791‐82	1:800
CD45	BV510	30‐F12	BioLegend	103137	1:200
LY6C	BV605	HK1.4	BioLegend	128036	1:300
CD11c	BV711	NK416	BioLegend	117349	1:200
MHCII	AF700	M5/114.15.2	BioLegend	107622	1:600

a All antigens listed are surface antigens.

### Materials

Cell suspensions (Basic Protocol 2 or 3)
Fc block (purified anti‐mouse CD16/CD32, clone S17011E; Biolegend, cat. no. 156604), diluted 1:100
FACS buffer (see recipe)
Phosphate‐buffered saline (PBS), sterile (BioConcept, cat. no. 3‐05F39‐I, or equivalent)
Fluorochrome‐coupled anti‐mouse monoclonal antibodies (BioLegend or equivalent; see Tables 1, 2, 3)
Fixation reagent (Cytofix/Cytoperm Fixation and Permeabilization Solution; BD Bioscience, cat. no. 554722)
Perm/Wash buffer (BD Bioscience, cat. no. 554723)
Distilled, deionized water (ddH_2_O)
Fluorescently labeled counting beads (Calibrite Beads, BD Bioscience, cat. no. 340486, or equivalent); see recipe for their preparation
5‐ml FACS tubes (BD/Falcon, cat. no. 7002120, or equivalent)
Tabletop centrifuge with swing‐out rotor (Heraeus Megafuge 16R or equivalent)
Vortex‐Genie 2 (Scientific Industries or equivalent)
5‐ml FACS tubes with cell strainer caps (BD/Falcon, cat. no. 7001431, or equivalent)
Flow cytometer (Cytoflex S or equivalent)

#### Surface staining of murine cLN and tongue cells for flow cytometric analysis

1 Prepare one 5‐ml FACS tube per sample for staining.
  Staining in FACS tubes is recommended over microtiter plates. Tongue cell suspensions contain abundant non‐immune cells and debris, and staining in tubes generally provides more consistent results than staining in plates.
2 Transfer cell suspension into 5‐ml FACS tubes and centrifuge 5 min at 560 × *g*, 4°C.
  Steps 3‐6 are required only when staining myeloid subsets. For other cell subsets, proceed to step 7.
3 Resuspend cells in 100 µl Fc block (diluted 1:100; purified anti‐mouse CD16/CD32, clone S17011E) in FACS buffer.
4 Incubate for 30 min at 4°C.
5 Wash samples by adding 1 ml FACS buffer and spinning down for 5 min at 560 × *g*, 4°C.
6 Remove the supernatant by carefully decanting it by hand and absorbing the remaining droplets with a wipe.
7 Prepare antibody mix (surface antibodies) in PBS: 100 µl per sample for whole tongue or tongue epithelium or 50 µl per sample for cLNs.
  Staining panels optimized for acquisition on a Cytoflex S are provided in Tables 1, 2, 3.
  If cell suspension from half a tongue or more is used, it is advisable to split the suspension to two 5‐ml FACS tubes for better antibody binding efficiency.
  Enzymatic digestion and mechanical disruption of the tissue can affect the integrity of certain cell surface epitopes and thus affect the antibody binding. For example, the CD4 clone RM4‐5 is not suitable in combination with collagenase IV or dispase II digestion. Therefore, antibody performance should be empirically validated under the specific digestion conditions used.
8 Add 50 µl (cLNs) or 100 µl (tongue) of the antibody mix per sample and mix well by pipetting up and down.
  The tongue pellet is very sticky and needs thorough up‐and‐down pipetting to resuspend well. Vigorous vortexing is not recommended. This also applies to all subsequent resuspension steps.
9 Gently vortex all FACS tubes for 3 sec at low speed.
10 Incubate for 30 min at 4°C in the dark.
11 Wash samples by adding 1 ml FACS buffer, centrifuging for 5 min at 560 × *g*, 4°C, and discarding the supernatant.
  - If same‐day acquisition is planned: Resuspend the pellet in 200 µl FACS buffer and proceed to step 19.
  - If same‐day acquisition is not possible: Proceed to step 12 and then to step 19.
  - If intracellular staining is required: Proceed to steps 13‐18.
12 Fix and permeabilize cells by adding 100 µl Cytofix/Cytoperm reagent per tube and mix by pipetting up and down. Incubate for 20 min at 4°C in the dark. Add 1 ml FACS buffer and centrifuge 5 min at 560 × *g*, 4°C. Discard the supernatant and resuspend the pellet in 200 µl FACS buffer by pipetting up and down.
  Stained and fixed cells can be stored at 4°C in the dark for up to 5 days before data acquisition.

#### Intracellular staining of murine cLN and tongue cells for flow cytometric analysis

13 Fix and permeabilize cells by adding 100 µl Cytofix/Cytoperm reagent per tube and mix by pipetting up and down. Incubate for 20 min at 4°C in the dark.
14 Wash cells by adding 1 ml of 1× BD Perm/Wash buffer (diluted 1:10 in distilled, deionized water) and centrifuging for 5 min at 560 × *g*, 4°C.
15 Add 100 µl of the intracellular antibody mix (e.g., anti‐IL‐17, diluted in BD Perm/Wash buffer) to each tube and mix by pipetting up and down.
16 Incubate 45 min to overnight at 4°C in the dark.
  The optimal incubation time and temperature depend on the antibody. Overnight incubation increases detection of low‐abundant targets.
17 Wash by adding 1 ml FACS buffer and centrifuging for 5 min at 560 × *g*, 4°C.
18 Resuspend in 350 µl FACS buffer.
  Stained or fixed and stained cells can be stored at 4°C in the dark for up to 5 days before data acquisition.

#### Acquisition of flow cytometry data

**Figure 2:**
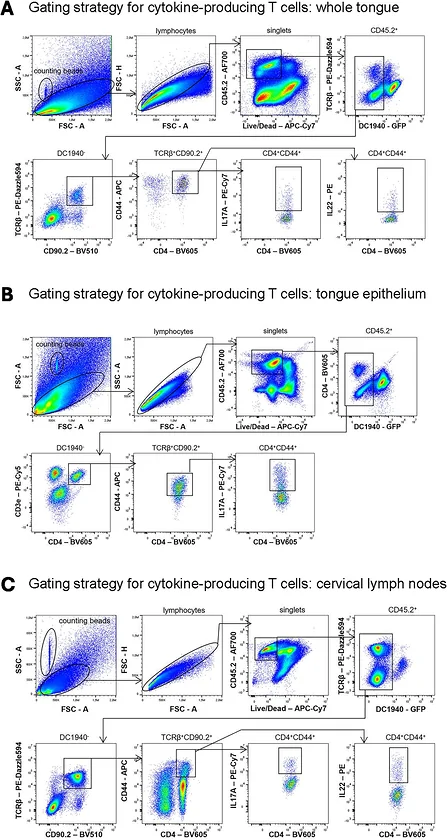
Gating strategy for cytokine‐producing CD4^+^ T cells in whole tongue, tongue epithelium and cervical lymph nodes of *C. albicans*‐colonized mice. Representative flow plots showing IL‐17A^+^ and IL‐22^+^ CD4^+^CD44^+^ T cells in whole tongue (**A**), tongue epithelium (**B**), and draining lymph node (**C**) on day 7 after *C. albicans* oral colonization.

**Figure 3:**
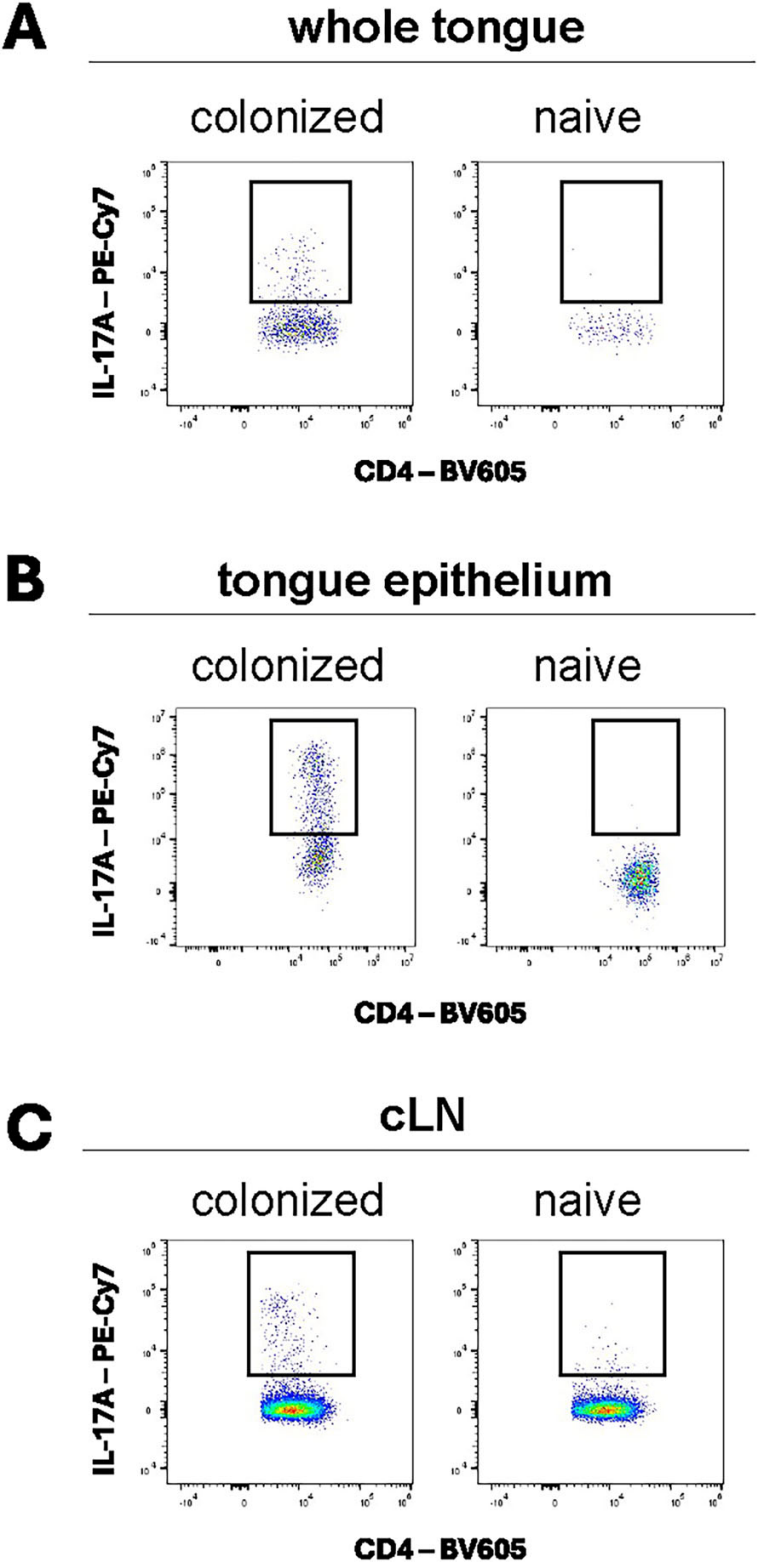
Representative flow plots comparing the population of IL‐17A^+^ CD4^+^CD44^+^ T cells in whole tongue (**A**), tongue epithelium (**B**), and draining lymph node (**C**) of naïve mice and mice that had been orally colonized with *C. albicans* for 7 days.

**Figure 4:**
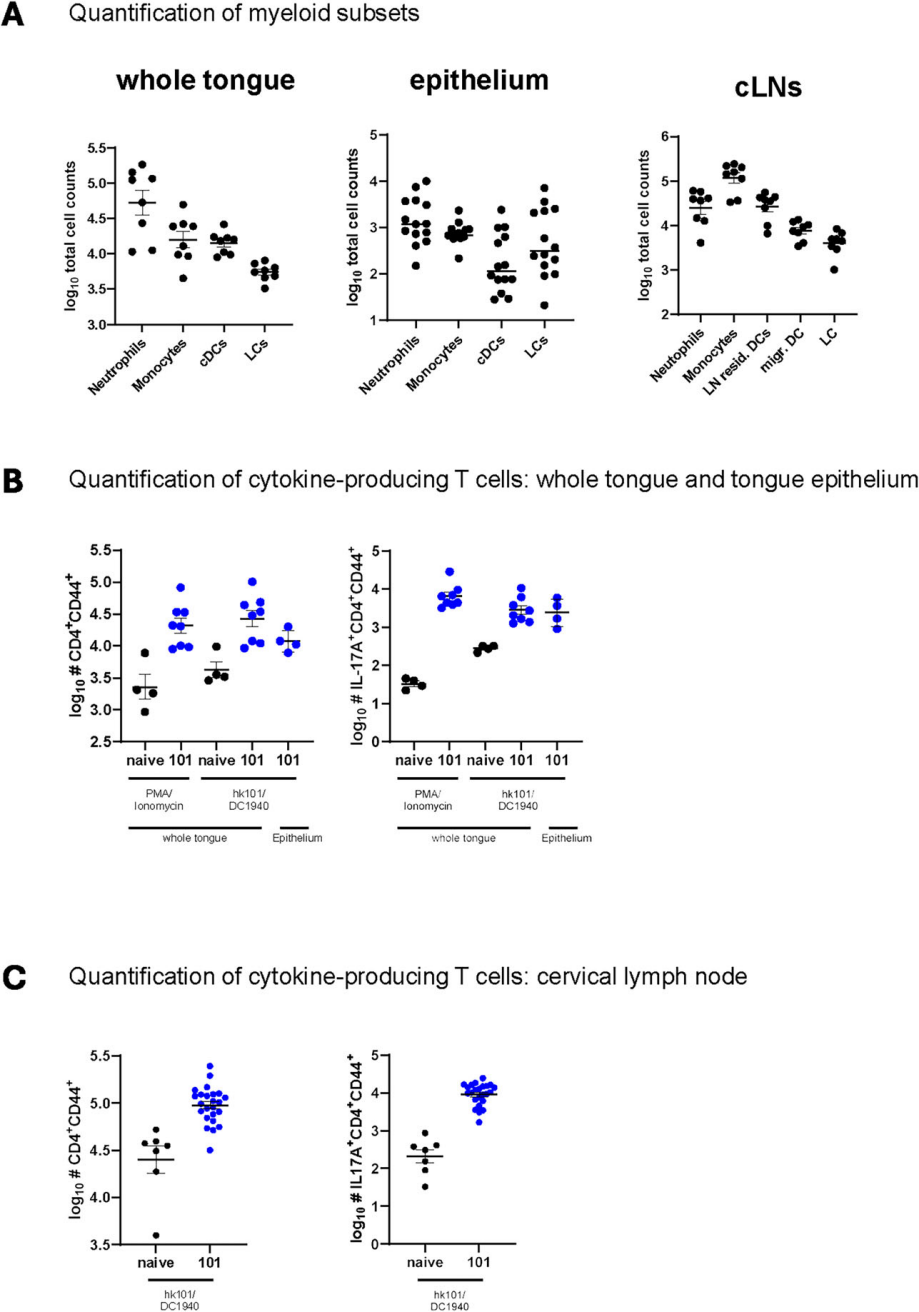
Quantification of myeloid and CD4^+^ T cells in whole tongue, tongue epithelium, and draining lymph nodes of *C. albicans*‐colonized mice. (**A**) Absolute counts of neutrophils, monocytes, DCs, and Langerhans cells (LCs) in whole tongue, tongue epithelium, and cervical lymph nodes (cLNs). (**B** and **C**) Absolute counts of total CD4⁺CD44⁺ and IL‐17A⁺CD4⁺CD44⁺ T cells in the tongue and tongue epithelium (**B**) and in the cervical lymph nodes (**C**) on day 7 after *C. albicans* oral colonization. Each symbol represents an individual mouse. Data are pooled from at least two independent experiments (mean ± s.e.m.).

19 Filter the sample into a FACS tube with cell strainer cap to remove any cell aggregates that may have formed during the staining process.
  Optionally, add 50 µl diluted counting beads per sample to allow determining absolute cell counts.
20 Acquire on a flow cytometer.
  For highly sensitive flow cytometers, applying an acquisition threshold can help reduce acquisition time when analyzing whole‐tongue single‐cell suspensions. Because whole‐tongue preparations contain large numbers of non‐immune cells and debris, event rates can become excessively high if acquisition is performed using only a scatter‐based threshold. To enrich acquisition of the relevant populations and minimize recording of irrelevant events, a fluorescence‐based threshold may be applied. A primary threshold on CD45 allows preferential selection of immune cells, while a secondary threshold can be set on the counting bead fluorescence channel (using an OR discriminator) to ensure that both CD45^+^ leukocytes and counting beads are properly recorded. If analyzing CD45^low^ immune cell subsets, epithelial cells, or other CD45^−^ stromal cells, samples should be acquired without CD45‐based thresholding, or alternative enrichment and gating strategies may be used, as appropriate.
  Acquisition and gating strategies should always be validated on the relevant instrument before analyzing experimental samples. It is advisable to include unstained samples to assess tissue autofluorescence, and appropriate single‐stain compensation controls and “fluorescence‐minus‐one” (FMO) controls to distinguish true signal from compensation spillover and to resolve closely related cell populations. Fully stained samples from naïve control mice should be included as biological negative controls to evaluate fungus‐induced qualitative and quantitative changes in specific cell populations. Acquisition threshold settings and gating decisions should be kept consistent across experimental groups wherever possible.
21 Analyze data as described by Cossarizza et al. (2019).
  Typically, live CD45^+^ leukocytes account for ∼1‐5% of total singlets in whole‐tongue single‐cell suspensions, ∼10‐15% in tongue epithelium single‐cell suspensions, and ∼60‐80% in cLN single‐cell suspensions prepared from C. albicans‐colonized mice. The number of distinct immune cell subsets that can be distinguished in a given sample depends on the complexity of the antibody panel, the instrument configuration, and the quality of the compensation matrix (Figs. 1, 2, 3, 4).
  Typically, 7 days after C. albicans colonization, ∼80‐90% of total tongue CD4^+^ T cells are activated, as indicated by CD44 positivity. Within the CD4^+^CD44^+^ population, 6‐20% of cells are IL‐17A^+^. In the cLNs, ∼10‐15% of CD4^+^ T cells are typically CD44^+^. Within this activated CD4^+^CD44^+^ population, 10‐20% of cells are IL‐17A^+^ (Fig. 4B‐C).
  The flow cytometry panels included in this protocol were designed for selected immune cell subsets. The panels may be adapted and/or expanded to incorporate additional antibodies for identifying other cell populations of interest, such as γδ T cells, innate lymphoid cells, neutrophils, macrophage and DC subsets, epithelial cells, etc.

## Support Protocol 1 EVALUATION OF FUNGAL BURDEN IN THE TONGUE OF *C. albicans*‐Colonized Mice

This protocol describes how to quantify fungal burden in the tongue after oral *C. albicans* colonization by plating tissue homogenates on yeast medium agar plates.

### Materials

0.05% NP‐40 buffer, sterile (see recipe)
YPD (+ ampicillin) agar plates (see recipe)

Laminar flow hood (Faster or equivalent)
2‐ml Safe‐Lock Eppendorf microcentrifuge tubes (Eppendorf, cat. no. 0030 123.344, or equivalent)
5‐mm steel balls (Berani Kugellager AG, cat. no. KU5 Niro 4034, or equivalent)
Precision balance reading to four decimal places (0.0001 g)
150 × 20‐mm petri dishes, sterile (Sarstedt, cat. no. 82.1184.500, or equivalent)
Type 20 surgical blades, sterile (Swann Morton, cat. no. 859221, or equivalent)
Curved dressing forceps (B. Braun Medical AG, cat. no. BD312R, or equivalent)
Dissecting scissors (Karl Hammacher GmbH, cat. no. 6075888, or equivalent)
TissueLyser II (Qiagen or equivalent)
Microbiological incubator, set to 30°C

#### Tongue tissue homogenization and plating

**Figure 5:**
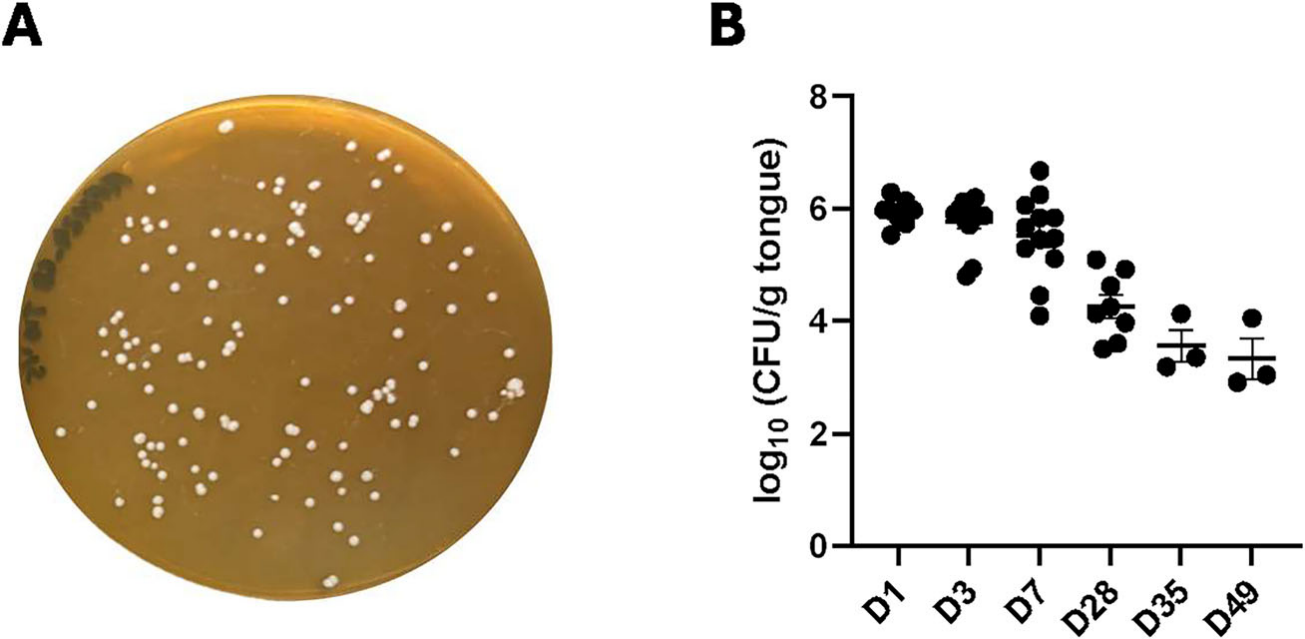
Evaluation of fungal burden in the tongue of *C. albicans*‐colonized mice. (**A**) Representative YPD agar plate with *C. albicans* colonies. (**B**) Quantification of CFU per gram tongue tissue on days 1, 3, 7, 28, 35, and 49 after oral colonization. Each symbol represents an individual mouse. Data are pooled from at least two independent experiments (mean ± s.e.m.).

1 Prepare one 2‐ml Safe‐Lock Eppendorf tube per tongue sample containing 500 µl of 0.05% NP‐40 buffer and one 5‐mm steel ball.
2 Measure the weight of the tube.
  Determining the weight of the tube before and after adding the tongue tissue makes it possible to calculate the weight of the tissue and, therefore, to normalize the number colony‐forming units (CFU) per gram of tissue.
3 Aseptically remove the tongue from euthanized mice as described in Basic Protocol 2, steps 17‐19.
  No transcardiac perfusion for fungal burden determination is required.
4 Cut the tongue longitudinally to secure at least one‐third of the tongue. Place it in the Eppendorf Safe‐Lock tube prepared in step 1.
5 Measure the weight of the tube, including tongue tissue.
6 Homogenize the tongue tissue using a TissueLyser II at 25 Hz for 3 min at room temperature.
  The tissue can be stored up to 4 hr at room temperature after harvesting and before homogenization.
  Visually inspect that the homogenization was successful.
7 Plate serial dilutions of homogenized tongue tissue on YPD (+ ampicillin) agar plates.
  For mice colonized with C. albicans strain 101 mice for 7 days, a 1:50 dilution by plating 10 µl from the total volume of 500 µl is recommended.
8 Incubate plates at 30°C for 24‐48 hr until colonies are easily visible.
  Plates can be transferred to 4°C for counting of CFUs at later time point.
9 Enumerate the number of colonies per plate and calculate the number of CFU per gram of tissue.
  Typically, the tongue fungal burden on day 1 after the colonization of mice with C. albicans strain 101 is ∼5.5‐6.5 log_10_ CFU/g tissue and declines thereafter, reaching stable levels at ∼3‐4 log_10_ CFU/g tissue by day 28 (Fig. 5).

## Support Protocol 2 VISUALIZATION OF TISSUE INTEGRITY, TISSUE INFLAMMATION, AND FUNGAL LOCALIZATION BY HISTOLOGY

This protocol describes how tongue tissue is processed for histology and periodic acid‐Schiff (PAS) staining to assess tissue integrity, tissue inflammation, fungal localization, and cellular infiltrations.

### Materials

Paraffin (Carl Roth, cat. no. CN49.1)
4% paraformaldehyde solution (Santa Cruz, cat. no. sc‐281692 or equivalent)
70%, 96%, and 99.8% ethanol (Reuss Chemie, cat. no. 7002106 [70%], and Sigma, cat. nos. 1.00971 [96%] and 02860‐1L [99.8%], or equivalent)
Hematoxylin and eosin staining solution
1% periodic acid solution (Carl Roth, cat. no. HP00.1, or equivalent)
Schiff's reagent (Carl Roth, cat. no. 36T9.1, or equivalent)
Mayer's Hemalum solution (Carl Roth, cat. no. T865.2, or equivalent)
Xylene (J.T. Baker, cat. no. 34105000PE, or equivalent)
Distilled water
Pertex mounting medium (Biosystem, cat. no. 41‐4012‐00, or equivalent)

Two pairs of curved dressing forceps (B. Braun Medical AG, cat. no. BD312R, or equivalent)
Dissecting scissors (Karl Hammacher GmbH, cat. no. 6075888, or equivalent)
1.5‐ml Safe‐Lock Eppendorf tubes (Eppendorf, cat. no. 7001817, or equivalent)
Microtome (Leica or equivalent)
Glass slides (Merck, cat. no. S4651, or equivalent)
Tissue cassettes (Leica, cat. no. LEIH38440300, or equivalent)
50‐ml Falcon tube (Sarstedt, cat. no. 7004175, or equivalent)
Cover slips (Faust, cat. no. 9161060, or equivalent)
Digital slide scanner (NanoZoomer 2.0‐HT, Hamamatsu, or equivalent)
NDP.view 2 software (Hamamatsu)

#### Fixing, processing, embedding, sectioning, and staining of tongue tissue samples

**Figure 6:**
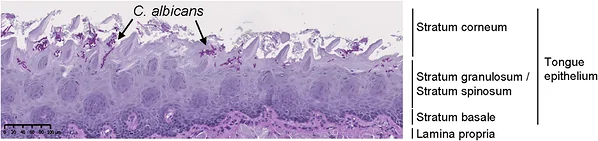
Histological analysis of the colonized tongue. Periodic acid‐Schiff (PAS)‐stained tongue sections from a *C. albicans*‐colonized animal. *C. albicans* appears brighter purple (arrows).

1 Remove the tongue from euthanized mice as described in Basic Protocol 2, steps 17‐19.
  No transcardiac perfusion is required for histological analysis.
2 Cut the tongue longitudinally with one precise cut to secure at least one‐third of the tongue.
  To get best quality it is important to cut the tissue with a single clean cut.
3 Place the tongue tissue into a 1.5‐ml Safe‐Lock Eppendorf tube containing 500 µl of 4% paraformaldehyde solution and incubate overnight at room temperature to fix the tissue.
4 Embed the tissue in paraffin.
5 Cut 9‐µm sagittal sections from the paraffin‐embedded blocks using a microtome and place on tissue slides.
6 Dewax the tissue sections by successive soaks in an ascending ethanol series: 2‐3 min in 70% ethanol, 2‐3 min in 96% ethanol, and 2‐3 min in 100% ethanol.
7 Rehydrate the tissue sections by successive soaks in a descending ethanol series: 2‐3 min in 100% ethanol, 2‐3 min in 96% ethanol, and 2‐3 min in 70% ethanol.
8 Wash tissue and stain sections with hematoxylin and eosin or with 1% PAS reagent.
9 Incubate for 3 min at room temperature.
10 Rinse with tap water for 10 min.
11 Rinse twice with distilled water for 2 min using a fresh Falcon tube.
12 Transfer slides into a fresh Falcon tube containing Schiff's reagent and incubate for 3 min at room temperature.
13 Rinse slide with warm tap water (at a minimum of 35°C) for 5 min.
14 Rinse with distilled water using a fresh Falcon tube.
15 Counterstain slide with Mayer's Hemalum solution for 5 min using a fresh Falcon tube.
16 Rinse slide in flowing tap water for 10 min.
17 Dehydrate slide section by successive soaks in an ascending alcohol series: 2 min in 70% ethanol, 2 min in 96% ethanol, and 2 min in 100% ethanol.
18 Clear slides in xylene for 3 min using a fresh Falcon tube.
19 Mount slides with Pertex, cover with a coverslip, and squeeze coverslip to distribute Pertex over the entire sample while removing any air bubbles.
20 Acquire high‐quality images with a digital slide scanner and analyze them using the NDP.view2 software.
  PAS‐positive cells appear rose to violet, and cell nuclei are stained in dark purple.
  Typically, low‐damage‐inducing isolates of C. albicans, such as strain 101, localize to the anucleated layer of the tongue epithelium (Frois‐Martins et al., 2025; Fig. 6).

## Support Protocol 3 EXTRACTION OF TOTAL RNA FROM THE TONGUE OF *C. albicans*‐COLONIZED MICE FOR RNA SEQUENCING AND RT‐qPCR

This protocol describes how tongue tissue is processed to obtain high‐quality RNA suitable for RNA‐sequencing and RT‐qPCR.

### Materials

RNase snap spray (Carl Roth, cat. no. A998.4, or equivalent)
Liquid nitrogen
TRI Reagent (Sigma, cat. no. T9424)
Absolute ethanol (Honeywell, cat. no 02860‐6×1L, or equivalent)
Direct‐zol RNA MiniPrep Plus with Zymo‐Spin IIICG Columns (capped; Zymo Research, cat. no. ZYM‐R2070)
DNase I kit (RNase‐Free DNase Set, Qiagen, cat. no. 7002201, or equivalent)
Ultrapure distilled water, DNase/RNase free (Invitrogen, cat. no. 7001621, or equivalent)

RNase‐free 5‐ and 2‐ml Safe‐Lock Eppendorf microcentrifuge tubes (Eppendorf, cat. nos. 7001817 and 0030 123.344)
Dissecting scissors (Karl Hammacher GmbH, cat. no. 6075888, or equivalent)
−80°C freezer
Dry ice
5‐mm steel balls (Berani Kugellager AG, cat. no. KU5 Niro 4034, or equivalent)
TissueLyser II (Qiagen) or equivalent
Microcentrifuge (Eppendorf model 5415D or equivalent)
TapeStation (Agilent or equivalent)

#### Extraction of RNA from murine tongue tissue

**Figure 7:**
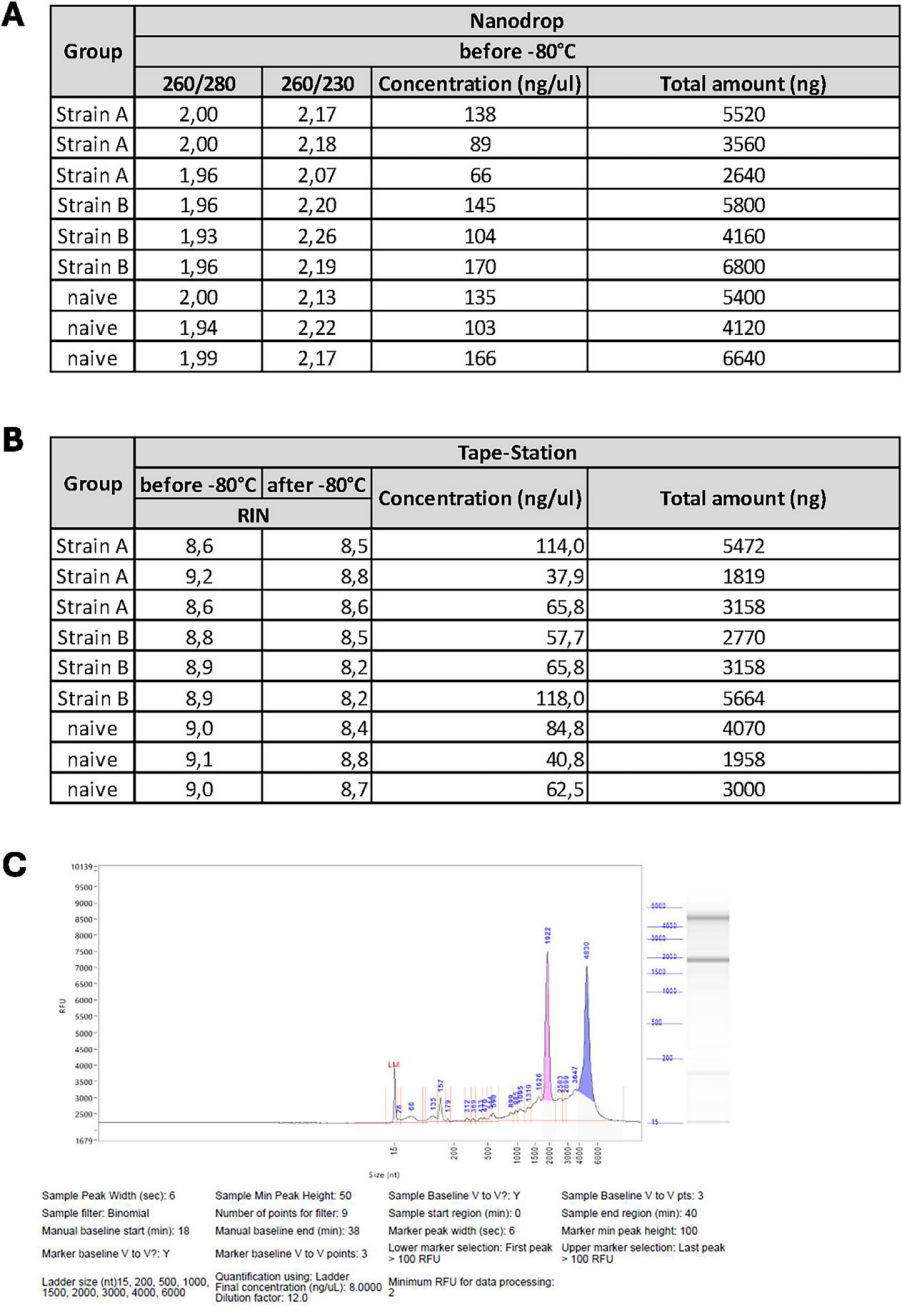
Quality control metrics for tongue RNA. RNA was isolated from the tongue of mice colonized with different *C. albicans* strains (strain A, strain B) or left uninfected (naïve). (**A**) RNA concentration as well as the *A*_260_/*A*_280_ and *A*_260_/*A*_230_ ratios were assessed by Nanodrop spectrophotometry. (**B**) RNA concentration and RNA integrity numbers (RIN) were assessed by TapeStation analysis before and after storage at −80°C. (**C**) Representative TapeStation electropherogram.

1 Remove the tongue from euthanized mice as described in Basic Protocol 2, step 16‐19.
  Performing a transcardiac perfusion is recommended to avoid isolating RNA from circulating cells.
2 Cut the tongue longitudinally to secure one‐half of the tongue and place it into a 2‐ml Safe‐Lock Eppendorf tube.
  In step 10, half of the tongue is divided into two samples and loaded onto two separate Zymo columns.
3 Using scissors thoroughly cleaned with RNase snap spray, cut the tongue tissue into small pieces inside the tube. Close the tube tightly.
4 Snap‐freeze in liquid nitrogen and store at −80°C until further processing.
5 Transfer tubes from −80°C to dry ice.
6 Add 1 ml ice‐cold TRI Reagent and a steel ball to each tube.
7 Homogenize the tissue using a TissueLyser II at 25 Hz for 6 min.
  Precool the adapter set, including the tube holder of the TissueLyser II, at −20°C for 10 min.
8 Put tubes on ice for 3 min.
9 Centrifuge in a microcentrifuge at full speed (21,300 × *g*) for 3 min at 4°C to sediment the debris.
10 Transfer twice 400 µl of the supernatant to two 2‐ml Safe‐Lock Eppendorf tubes.
  Carefully aspirate the supernatant to avoid disturbing or removing any debris.
11 Add 400 µl absolute ethanol and mix well by pipetting up and down.
12 Transfer the total content of each tube into a Zymo‐Spin column fitted onto a collection tube.
13 Follow the standard protocol of the Direct‐zol RNA MiniPrep Plus kit, including the DNase digestion step.
14 At the end, elute the RNA from the Zymo column with 25‐50 µl ultrapure DNase/RNase‐free water. Rest the column for 5 min at room temperature and then centrifuge 1 min at 12,000 × *g*, room temperature. Remove the column and store the tube containing the pure RNA on ice.
15 Remove a small aliquot of RNA for measuring RNA concentration and quality on the TapeStation.
16 Store the RNA at −80°C.
  Avoid freeze‐thaw cycles, which will impact the quality of the RNA.
  Typically, RNA of very high quality (RNA integrity number >8.0) is obtained with this protocol, which is suitable for RNA sequencing. If downstream applications do not require such a high RNA quality, RNA may also be isolated with TriReagent following the standard protocol without the Direct‐zol RNA MiniPrep Plus kit (Fig. 7).

## REAGENTS AND SOLUTIONS

### Brefeldin A solution, 55 µg/ml

To prepare a 5 mg/ml stock solution, resuspend 25 mg brefeldin A (Sigma Aldrich, cat. no. B6542) in 5 ml absolute ethanol. Store in 50‐µl aliquots at −20°C and avoid exposure to more than 10 freeze‐thaw cycles.

To prepare 1 ml working solution at 55 µg/ml, mix 11 µl brefeldin A with 989 µl complete R10.

### *C. albicans*, heat killed

Prepare the fungal suspension as described in Basic Protocol 1, steps 1‐9. Prepare a solution containing 5 × 10^7^ *Candida albicans* cells/ml in PBS. Heat kill in a heating block or water bath at 85‐95°C for 45 min. Store up to 6 months at 4°C.

### Collagenase I/DNase I digestion mix for cLNs

To prepare a 240 mg/ml collagenase I stock solution, resuspend 1 g collagenase I (Gibco/ThermoFisher, cat. no. 17100‐017) in 4.17 ml sterile PBS by gently mixing. Store in 200‐µl aliquots at −20°C and avoid exposure to more than 10 freeze‐thaw cycles.

To prepare a 20 mg/ml stock of DNase I, resuspend 100 mg DNase I (Sigma Aldrich, cat. no. DN25) in 5 ml sterile PBS by gently mixing. Store in 200‐µl aliquots at −20°C and avoid exposure to more than 10 freeze‐thaw cycles.

To prepare the collagenase I/DNase I digestion mix, combine 10 µl collagenase I stock and 10 µl DNase I stock per sample (prepare 20 µl per sample).

### Collagenase II/DNase I digestion mix for tongue epithelium

Resuspend 1 g collagenase II (Worthington, cat. no. NC9693955) in 5 ml PBS (200 mg/ml) by mixing gently. Store in 200‐µl aliquots at −20°C and avoid exposure to more than 10 freeze‐thaw cycles.

To prepare the collagenase II/DNase I digestion mix, combine 10 µl collagenase II stock and 5 µl DNase I stock in 985 µl RPMI 1640 per sample (prepare 1 ml per sample).

### Collagenase IV/DNase I digestion mix for whole tongue

To prepare a 240 mg/ml collagenase IV stock solution: Resuspend 1 g collagenase IV (Gibco/ThermoFisher, cat. no. 17104‐019) in 4.17 ml sterile PBS by gentle mixing. Store in 200‐µl aliquots at −20°C and avoid exposure to more than 10 freeze‐thaw cycles.

To prepare a 20 mg/ml stock of DNase I, resuspend 100 mg DNase I (Sigma Aldrich, cat. no. DN25) in 5 ml sterile PBS by gentle mixing. Store in 200‐µl aliquots at −20°C and avoid exposure to more than 10 freeze‐thaw cycles.

To prepare the collagenase IV/DNase I digestion mix, combine 10 µl collagenase IV stock and 10 µl DNase stock per sample. Prepare 20 µl per sample.

### Complete IMDM medium

Mix:
500 ml IMDM with glutamine, with 25 mM HEPES (Milian, cat. no. L0191‐500)
50 ml fetal calf serum (FCS; BioConcept, cat. no. 2‐01F10‐I; 8.8% [v/v] final)
0.5 ml of 50 mM β‐mercaptoethanol (Gibco, cat. no. 31350010; 44 µM final)
5 ml penicillin‐streptomycin (Amimed, cat. no. 4‐01F00‐H; both 0.88% [v/v] final)
Store up to 8 weeks at 4°C.

### Complete R10 medium

Mix:
500 ml RPMI 1640 with l‐glutamine (Life Technologies, cat. no. 7001611, or equivalent)
50 ml FCS (BioConcept, cat. no. 2‐01F10‐I; 8.8% [v/v] final)
5 ml penicillin (10,000 IU/ml)‐streptomycin (10 mg/ml) (Amimed, cat. no. 4‐01F00‐H; 0.88% [v/v] final)
5.5 µl of 50 mM β‐mercaptoethanol (Gibco, cat. no. 31350010; 44 µM final)
5 ml of 1 M HEPES (Gibco, cat. no. 15630056; 8.76 mM final)
5 ml of 100× nonessential amino acids (Gibco, cat. no. 11140035; 0.88% [v/v] final)
5 ml of 100 mM sodium pyruvate (Gibco, cat. no. 11360070; 0.88 mM final)
Store up to 8 weeks at 4°C.

### Dispase II digestion mix for tongue epithelium

Resuspend 1 g dispase II (Roche, cat. no. 4942078001) in 50 ml PBS (20 mg/ml) by mixing gently. Store in 500‐µl aliquots at −20°C and avoid exposing to more than 10 freeze‐thaw cycles. To prepare the dispase II digestion mix, combine 50 µl of dispase II stock in 450 µl RPMI 1640 per sample (prepare 500 µl per sample). Prepare fresh before use.

### EDTA, 5 mM

Mix 500 µl of 0.5 MEDTA stock solution, pH 8 (Invitrogen, cat. no. AM9260G) with 50 ml PBS (BioConcept, cat. no. 3‐05F39‐I, or equivalent). Store up to several months at 4°C.

### FACS buffer

Mix:
100 ml of 10× PBS (see recipe; 1× final)
10 ml decomplemented FCS (BioConcept, cat. no. 2‐01F10‐I; 1% [v/v] final)
10 ml of 0.5 M EDTA (Invitrogen, cat. no. AM9260G; 5 mM final)
2 ml of 500× NaN_3_ (Bioworld, cat. no. 41920044‐2; 1× final)
880 ml distilled, deionized water (Milli‐Q grade)
Store up to 8 weeks at 4°C.

### Fluorescently labeled counting beads

Dilute 1‐2 drops of fluorescently labelled counting beads (Calibrite Beads, BD Bioscience, cat. no. 340486, or equivalent) in 5 ml PBS. Mix well by vortexing. Count the number of beads per 50 µl using a hemocytometer; aim for 2,000‐5,000 beads per 50 µl. Store up to 3 months at 4°C.

### Ketamine/xylazine mix

Mix:
325 µl ketamine (100 mg/ml; Streuli, cat. no. 6586716, or equivalent)
325 µl xylazine (20 mg/ml; Streuli, cat. no. 6586900, or equivalent)
4.35 ml 1× PBS
Prepare fresh each time.

### MACS buffer

Mix:
500 ml PBS (BioConcept (NEB), cat. no. 3‐05F39‐I, or equivalent)
5 ml of 1% (v/v) FCS (BioConcept, cat. no. 2‐01F10‐I)
2 ml of 0.5 M EDTA, pH 8 (Invitrogen, cat. no. 10135423; 2 mM final)
Store up to 8 weeks at 4°C.

### NP‐40, 0.05%

Mix 0.25 ml Nonidet P‐40 (NP‐40; AppliChem, cat. no. A1694,0250) with 500 ml distilled, deionized water. Sterile filter through a 0.22‐µm‐pore‐size syringe filter. Store up to several months at room temperature.

### Phosphate‐buffered saline (PBS), 10×

Dissolve in 800 ml H_2_O:
17.8 g Na_2_HPO_4_ (Merck/Sigma, cat. no. 71640)
2.4 g KH_2_PO_4_ (Merck, cat. no. 1.04873.0250)
80 g NaCl (Merck/Sigma, cat. no. S9888)
2 g KCl (Merck/Sigma, cat. no. 1711999)
Adjust pH to 7.4 with 1 N HCl
Add H_2_O to 1 liter
Autoclave
Store up to several months at room temperature.

### PMA/ionomycin

To prepare a 1 mg/ml stock of ionomycin, resuspend 1 mg ionomycin calcium salt from *Streptomyces conglobatus* (Sigma, cat. no. I0634) in 1 ml absolute ethanol. Store in 20‐µl aliquots at −20°C and avoid exposure to more than 10 freeze‐thaw cycles.

To prepare 1 mg/ml phorbol 12‐myristate 13‐acetate (PMA), resuspend 1 mg PMA (Sigma‐Aldrich, cat. no. P8139) in 1 ml absolute ethanol. Store in 10‐µl aliquots at −20°C and avoid exposure to more than 10 freeze‐thaw cycles.

To prepare 1 ml PMA/ionomycin working solution, mix 0.1 µl PMA stock, 1 µl ionomycin stock, and 999 µl complete R10 medium.

### Trypan blue

Prepare a 0.4% trypan blue working solution by mixing 1 ml of 4% trypan blue stock solution (Sigma, cat. no. T8154) with 9 ml PBS. Store up to several months at room temperature.

### YPD agar plates with or without ampicillin

Mix:
20 g d‐(+)‐glucose monohydrate (Sigma, cat. no. 49159; 2% [w/v] final)
10 g Bacto Yeast extract (BD, cat. no. 212720; 1% [w/v] final)
20 g Bacto Peptone (BD/Gibco, cat. no. 211820; 2% [w/v] final)
20 g agar (Sigma, cat. no. A1296; 2% [w/v] final)
Bring up to a final volume of 1 L with distilled, deionized water (Milli‐Q grade). Add a magnetic stir bar. Autoclave. Cool down to 50‐55°C while stirring on a magnetic plate for ∼1 hr.

*Optionally*: Add 1 ml ampicillin (100 µg/ml in PBS, sterile filtered through a 2‐µm‐pore‐size filter and stored at −20°C; AppliChem, cat. no. A0839,0100; 0.1 mg/liter final).

Pour into ∼15 ml per dish into petri dishes. Dry at room temperature overnight. Store up to several weeks at 4°C.

### YPD liquid medium

Mix:
20 g d‐(+)‐glucose monohydrate (Sigma, cat. no. 49159; 2% [w/v] final)
10 g Bacto Yeast extract (BD, cat. no. 212720; 1% [w/v] final)
20 g Bacto Peptone (BD/Gibco, cat. no. 211820; 2% [w/v] final)

Bring up to a final volume of 1 liter with distilled, deionized water (Milli‐Q grade). Autoclave. Store up to several weeks at 4°C.

## COMMENTARY

### Critical Parameters

#### Biological variables influencing oral Candida albicans colonization

Several host and environmental variables can influence oral *C. albicans* colonization and should be considered during study design. Host age affects epithelial barrier integrity and immune function, whereas sex‐dependent differences in innate and adaptive immunity may alter fungal persistence and host responses. We therefore recommend using age‐matched mice and either a single sex or balanced numbers of males and females, with sex incorporated into the experimental design. The composition of the resident microbiota is another important determinant of colonization efficiency and immune homeostasis. To reduce microbiota‐associated variability, mice should be sourced from the same vendor, maintained under standardized husbandry conditions, and cohoused prior to experimentation whenever possible. When using genetically modified mice, littermate controls should be used. Although randomizing cage allocation is part of a good study design, it is not advised in case of oral *C. albicans* colonization. The fungus disseminates rapidly along the entire gastrointestinal tract and can be detected in the faces within hours after sublingual administration. Due to the coprophagic behavior of mice, naïve animals become colonized with *C. albicans* when kept together in a cage with colonized mice. Attention to these biological variables will improve experimental reproducibility and facilitate comparison of results across laboratories.

#### Influence of mouse and fungal strain background on the outcome of C. albicans oral colonization

The genetic background of both the host and the fungus can profoundly influence the experimental outcome of oral colonization. Mice with different genetic backgrounds vary in their susceptibility to oral colonization and infection because of inherent differences in innate and adaptive immunity, as early studies have shown (Elahi et al., 2001, 2000). Likewise, *C. albicans* isolates display considerable genetic and phenotypic diversity that affects traits including filamentation, epithelial adhesion, invasion, stress tolerance, immune activation, and persistence within the oral mucosa (Frois‐Martins et al., 2025; Lemberg et al., 2022; Schonherr et al., 2017). Consequently, colonization levels, tissue pathology, and host immune responses may vary substantially depending on the combination of mouse and fungal strains used. To facilitate comparison between studies, investigators should employ well‐characterized mouse and fungal strains, minimize genetic variation within experiments, and report strain identities and relevant genetic modifications in sufficient detail.

Importantly, the methodological workflow described in this protocol, including oral fungal administration, tissue processing, and flow cytometry analyses as well as fungal burden, histology, and RNA‐based readouts, can be applied independently of the host and fungal strain background, as long as host‐ and strain‐dependent differences are taken into account during experimental design and data interpretation. The methods were established with C57BL/6 mice kept under SPF husbandry conditions and with the low‐damage‐inducing *C. albicans* strain 101. This low‐damage‐inducing strain establishes colonization in the outermost keratinized layer of the tongue epithelium within hours after sublingual administration (Frois‐Martins et al., 2025). The total tongue fungal burden declines over the first 2 weeks and then remains stable over several months (Kirchner & LeibundGut‐Landmann, 2021). Innate immune activation by strain 101 is very limited, while CD4^+^ T cell immunity is strongly activated, with *C. albicans*‐specific Th17 cells residing in the tongue (Kirchner & LeibundGut‐Landmann, 2021; Kirchner et al., 2019; Schonherr et al., 2017). Several other strains exhibit similar behavior in the murine oral cavity (Frois‐Martins et al., 2026; Schonherr et al., 2017). In contrast, the high‐damage‐inducing strain SC5314 accesses deeper epithelial layers, elicits an acute inflammatory response—characterized by massive infiltration of inflammatory neutrophils and monocytes—and in turn is rapidly cleared from the host (Conti et al., 2009; Schonherr et al., 2017). Despite the short kinetics, strain SC5314 also induces a prominent type 17 response, in which diverse innate‐like T cells contribute to cytokine production (Conti et al., 2014; Gladiator et al., 2013; Sparber et al., 2018). Other *C. albicans* strains may behave like one of the two mentioned strains or manifest a different behavior again. This illustrates the need to adjust analysis time points and the focus on specific immune cell types in a context dependent manner.

#### Quality of the single‐cell suspension

A high‐quality single‐cell suspension is essential for reliable phenotypic and functional assessment of tongue immune cell subsets. Good sample quality ensures efficient recovery of viable cells while minimizing cell aggregates, debris, and other artifacts that can compromise downstream analyses. A well‐prepared suspension therefore improves the resolution of immune cell populations, increases reproducibility, and enables more robust interpretation of both surface phenotype and functional readouts.

Although enzymatic digestion of tongue tissue is required to obtain high‐quality single‐cell suspensions for flow cytometric analysis, it may also introduce technical biases, including reduced recovery of selected cell populations, diminished cell viability and loss of digestion‐sensitive antibody epitopes. Therefore, antibodies should be validated under the specific digestion conditions used. Mitigation strategies include the use of alternative antibody clones, fluorochrome combinations, and staining strategies. Digestion conditions, including enzyme concentration, incubation time, temperature, and mechanical dissociation techniques, should be kept consistent across experimental groups within an experiment and across independent experiments to maximize reproducibility.

#### Flow cytometry compensation

Tongue samples typically exhibit substantial autofluorescence. Therefore, automatically calculated compensation must be carefully verified and, if necessary, manually adjusted based on fluorescence minus one (FMO) controls and empirical experience with tongue tissue. Particular attention should be given to markers that are expressed at low levels, to dim fluorochromes, and to fluorochromes detected in channels that are prone to signal spillover or high background. The exclusion of dead cells by means of viability dyes is highly recommended as dead cells markedly increase nonspecific background and autofluorescence.

Although single‐cell suspensions of tongue epithelium and cLNs have a lower intrinsic autofluorescence than whole tongue samples, the same principles apply. Compensation settings should be carefully checked for every single experiment to ensure accurate resolution of fluorochrome spillover and correct identification of cellular subsets.

#### Translational considerations and limitations of the mouse model

Although the mouse model of *C. albicans* oral colonization recapitulates many key features of host‐fungal interactions at the oral mucosa in humans, several limitations should be considered when extrapolating findings. Differences in the composition of the oral microbiota, oral anatomy, salivary physiology, and epithelial biology may influence fungal colonization dynamics and immune responses. Furthermore, colonization is established experimentally in adult mice under standardized laboratory conditions, which cannot fully reproduce the complex environmental exposures, microbial communities, and host heterogeneity that shape natural colonization in humans from shortly after birth. Although many fundamental mechanisms of mucosal antifungal immunity, including IL‐17‐dependent host defense, are highly conserved between mice and humans, species‐specific differences in immune regulation and epithelial responses should be considered when interpreting results. Consequently, observations obtained using this model should ideally be complemented by studies using primary human cells or tissues, along with analyses of patient samples, to establish their relevance to human oral candidiasis and asymptomatic *C. albicans* carriage.

#### Expansion of the methodological platform

The workflow is not limited to the readouts described in this protocol. Single‐cell sequencing, spatial transcriptomics, multiplex imaging, and fungal transcriptomics may be pursued to resolve immune and epithelial cell heterogeneity in the *C. albicans*‐colonized tongue, to link antifungal responses to fungal localization, and to investigate fungal adaptation during colonization. In addition, the oral microbiome and mycobiome may be assessed to monitor for dysbiosis and explore bacterial‐fungal interactions.

### Author Contributions

**Julia Lagler**: Conceptualization; methodology; investigation; formal analysis; visualization; writing—original draft; writing—review and editing; data curation. **Mathieu Courivaud**: Methodology; investigation; data curation; visualization; writing—review and editing; formal analysis. **Johnny Bonnardel**: Methodology; investigation; supervision; funding acquisition; project administration; writing—review and editing; validation; resources. **Salomé LeibundGut‐Landmann**: Conceptualization; data curation; validation; supervision; funding acquisition; project administration; resources; writing—original draft; writing—review and editing.

### Conflict of Interest

The authors declare not conflict of interest.

## Data Availability

The data, tools, and material (or their source) that support the protocol are available from the corresponding author upon reasonable request.
